# Emergency transportation for acute alcohol intoxication four years after the coronavirus disease 2019 pandemic: a retrospective observational study

**DOI:** 10.1265/ehpm.24-00182

**Published:** 2024-10-08

**Authors:** Marina Minami, Natsuko Nakamura, Masamitsu Eitoku, Atsufumi Kawauchi, Takeshi Murakami, Narufumi Suganuma, Kingo Nishiyama, Masato Miyauchi

**Affiliations:** 1 Integrated Center for Advanced Medical Technologies (ICAM-Tech), Kochi Medical School Hospital, Kochi 783-8505, Japan; 2 Department of Environmental Medicine, Kochi Medical School, Kochi University, Nankoku, Kochi 783-8505, Japan; 3 Department of Health Policy, Kochi Prefectural Government, Kochi City, Kochi 780-8570, Japan; 4 Department of Disaster and Emergency Medicine, Kochi Medical School, Nankoku, Kochi 783-8505, Japan

**Keywords:** Emergency transportation, COVID-19, Acute alcohol intoxication

## Abstract

**Background:**

In a study conducted in Kochi Prefecture, Shikoku, Japan, during the early stages of the pandemic in spring 2020, we found that emergency transportations due to acute alcohol intoxication decreased. We aimed to determine how the decline in the number of emergency transportations due to acute alcohol intoxication changed during the four years following the COVID-19 pandemic’s onset.

**Methods:**

This study used data of 107,013 emergency transportations from the Kochi-Iryo-Net database, Kochi Prefecture’s emergency medical and wide-area disaster information system. We categorized emergency transportation cases according to the diagnoses entered into the system by the attending physician, which were then divided into alcohol- and non-alcohol-related intoxication cases based on the diagnostic codes in the International Classification of Diseases Manual, 10th edition, Clinical Modification. We performed chi-square tests and multiple logistic regression to examine the association between emergency transportations and acute alcohol intoxication.

**Results:**

The number of emergency transportations due to acute alcohol intoxication was 412 (1.8%) in 2019, and it declined to 268 (1.4%), 248 (1.2%), 270 (1.2%), and 283 (1.3%) in 2020, 2021, 2022, and 2023, respectively. After adjusting for confounding factors such as fire department and age, a significant decrease was observed in the subsequent years compared with 2019 (2020: adjusted odds ratio, 0.79; 95% confidence interval, 0.68–0.93; 2021: adjusted odds ratio, 0.74; 95% confidence interval, 0.63–0.87; 2022: adjusted odds ratio, 0.73; 95% confidence interval, 0.62–0.85; 2023: adjusted odds ratio, 0.76; 95% confidence interval, 0.65–0.89).

**Conclusions:**

This study examined changes in emergency transportation due to acute alcohol intoxication during and after the COVID-19 pandemic, especially when social events and other activities returned to “normal.” Compared with 2021, which was when emergency transportations due to acute alcohol intoxication were at their lowest, a slight increase was observed in the number of transportations in subsequent years.

## Background

The first cases of coronavirus disease 2019 (COVID-19) were reported in December 2019 in Wuhan, China, and were relatively quickly followed by the beginning of the COVID-19 pandemic in early 2020 [1]. This pandemic has changed people’s behaviors, including their drinking. For example, a review of the effects of COVID-19 on adolescents reported an increase in alcohol consumption frequency [2, 3], while another review, now on changes in substance and alcohol use during the COVID-19 pandemic, showed a clear trend toward increased alcohol consumption and a higher use of other substances [2, 3]. Mental health factors were the most common correlates or triggers of increased use of both alcohol and other substances in a previous study [2]. In Europe, a report that measured changes in overall alcohol use found that slightly more people who showed a decrease (versus an increase) in alcohol use during the pandemic reduced their temporary, heavy episodic drinking behaviors [4]. In Japan, an investigation into shifts in alcohol consumption and the correlated psychosocial aspects during the COVID-19 pandemic revealed a connection of severe alcohol-related problems with heightened psychological issues, workplace and academia challenges, and economic hardships [5]. Generally, these past lines of evidence show that acute alcohol intoxication trends during the COVID-19 pandemic may have varied by one’s original drinking habits and social context.

Acute alcohol intoxication is a clinically harmful condition that typically occurs following the ingestion of a large amount of alcohol in a short period [6, 7]. It is characterized by a range of behavioral and neurological symptoms, including impaired judgment, mood changes, loss of coordination, and slurred speech, and severe cases may experience respiratory depression, coma, and cardiac arrest [7]. Intoxication severity is directly related to the blood alcohol concentration, albeit regular drinkers often develop tolerance and show fewer signs of intoxication at blood alcohol concentrations that would significantly impair non-regular drinkers [8].

An important association has existed between events, festivals, and alcohol consumption since ancient times. Indeed, higher alcohol consumption due to holidays and social and sporting events increases alcohol-related patient visits, making these situations a risk for patient influx into the emergency department (ED) [9]. In a study focusing on drug- and alcohol-related symptoms and the impact of massive sporting events on local health services, alcohol consumption contributed to ambulance transport cases from the event site [10]. In instances wherein individuals have been admitted to the ED because of heavy drinking, there have been accounts of alcohol-related aggression within the ED [11]. People with injuries related to violence who test positive for blood alcohol upon admission frequently disclose pre-event alcohol consumption, exhibit a greater frequency of heavy drinking, and report more alcohol-related problems than those admitted for injuries unrelated to violence [12]. In summary, drinking behavior can easily be influenced by external interactions with others in situations such as events and drinking parties, and it can also be impacted by the atmosphere of the event.

Following the COVID-19 outbreak, various events in Japan had to be cancelled. Still, wearing masks—which were supposed to be worn indoors in principle to prevent infection—in the country was not mandatory in respect of individuals’ independent choice, and it became a matter of personal judgment after March 13, 2023 [13]. Then, on May 3, 2023, the infection status of COVID-19 under the Infectious Disease Control Law became Category 5 [14]. Specifically, the Japanese government stated that it would “not uniformly require basic infection control measures in daily life;” that the “Infectious Disease Control Law no longer requires persons who are positive for the new coronavirus and those who have been in close contact with it to refrain from leaving their homes;” and that “Medical examinations will be available at a wide range of medical facilities, instead of being available only at a limited number of medical facilities” [14]. Thus, the situation surrounding the COVID-19 pandemic gradually changed throughout the four years following its onset, and people’s related attitudes and customs have slowly evolved.

Kochi Prefecture, comprising 37.8% of Shikoku’s total area and 1.9% of Japan’s total area, ranks as the 18th largest prefecture in the country. The Prefecture is nestled in the southern half of Shikoku, has a fan-shaped topography facing the Pacific Ocean, and boasts a distinct cultural landscape. According to the Kochi Prefectural Government’s website, its citizens are known for their sociable nature, easily befriending others over drinks (mostly alcoholic), and their warm-hearted and kind disposition toward everyone [15]. Renowned nationally as a haven for sake enthusiasts young and old, Kochi Prefecture has a vibrant drinking culture. Local sake, cuisine, and traditional games add to the convivial atmosphere, making drinking an enjoyable experience in the Prefecture [16]. Before the onset of COVID-19, approximately 400 emergency transportations occurred yearly in Kochi Prefecture, with 1.1% being attributed to acute alcohol intoxication [17]. Meanwhile, in the year preceding the onset of COVID-19 in Tottori Prefecture, the number of emergency transportations was lower (230 recorded cases), it decreased further in 2020 (165 recorded cases), and accounted for 0.7% of the total number of transportations during the pandemic year [18]. Despite these two prefectures having similar population sizes, Kochi Prefecture had significantly more emergency transportations. At the same time, research aimed at identifying trends regarding acute alcohol intoxication in areas with unique drinking cultures, like Kochi Prefecture, may provide clues to reducing emergency transportations due to such forms of intoxication.

Furthermore, according to the numbers published by the Tokyo Fire Department on emergency medical evacuations due to acute alcohol intoxication, the figures are increasing, with more than 10,000 people being transported to hospitals for such intoxication annually. Specifically, when considering the last 5 years, the number reached its peak in 2019, when more than 18,000 people were transported to hospitals for acute alcohol intoxication [19]; in spring 2020, when the general public’s self-restraint to prevent the spread of COVID-19 became stronger, this number decreased [17], and an accentuation in the decline could be observed in 2022 [20, 21]. That is, while the figures were generally increasing in the last decade, during periods characterized by higher self-restraint in the Japanese population, the number of hospitalizations due to acute alcohol intoxication decreased significantly, possibly because of the reduced hours of operation of restaurants and bars to prevent infection [18].

Thereafter, for the first time after the onset of COVID-19, spring 2023 brought with it the resumption of numerous social events. As the previous restrictions were lifted, people regained the opportunity to socialize and partake in drinking. This study aimed to determine how the trend of decline in emergency transportations due to acute alcohol intoxication evolved over the four years following the onset of the COVID-19 pandemic.

## Methods

### Study design and setting

Japan has a nationwide combined fire-and-ambulance service funded by taxpayers and available free of charge to anyone, anytime, and anywhere through an emergency phone number (i.e., 119) [1]. Local governments provide ambulance services through their fire departments, whereas municipal governments oversee fire department management. National (Fire and Disaster Management Agency, the Ministry of Internal Affairs and Communications) and prefectural governments and agencies provide guidance and advice to municipal fire departments [1].

We conducted the present study in Kochi City, the prefectural capital of Kochi Prefecture, which in turn is located on the southern side of the island of Shikoku, Japan. As of 2021, Kochi Prefecture had 15 fire departments, with one fire-and-ambulance headquarters and 10 combined fire-and-ambulance stations within the city. The Kochi-Iryo-Net database was established as part of the emergency medical and disaster information system for Kochi Prefecture [17, 22]. Whenever a fire department dispatches an ambulance and its crew, information regarding the dispatch (e.g., fire department and date and time of call) is recorded in the Kochi-Iryo-Net database. Upon the patient’s arrival at the medical institution, nurses and the attending physician record information pertinent to the case (e.g., degree of severity and disease classification) in the institution’s system for medical record management purposes. This information is later aggregated into the Kochi-Iryo-Net database according to the destination medical institution’s data in the Kochi prefectural government. For this retrospective study, we employed these aggregated data.

Patients transported by ambulance were included in this study according to the information in the Kochi-Iryo-Net database. This study did not include patients who were not registered in the database, and patients with missing data were excluded. As mentioned in the last paragraph, after the emergency transportation reaches the medical institution, the attending physician in charge enters information about the case in the institution’s system, having the possibility of entering up to three diagnoses based on a list of injuries available in the system. In this study, we categorized ED visit cases according to the diagnostic codes in the International Classification of Diseases Manual, 10th edition, Clinical Modification, distinguishing between cases of alcohol- and non-alcohol-related intoxication.

**Fig. 1:**
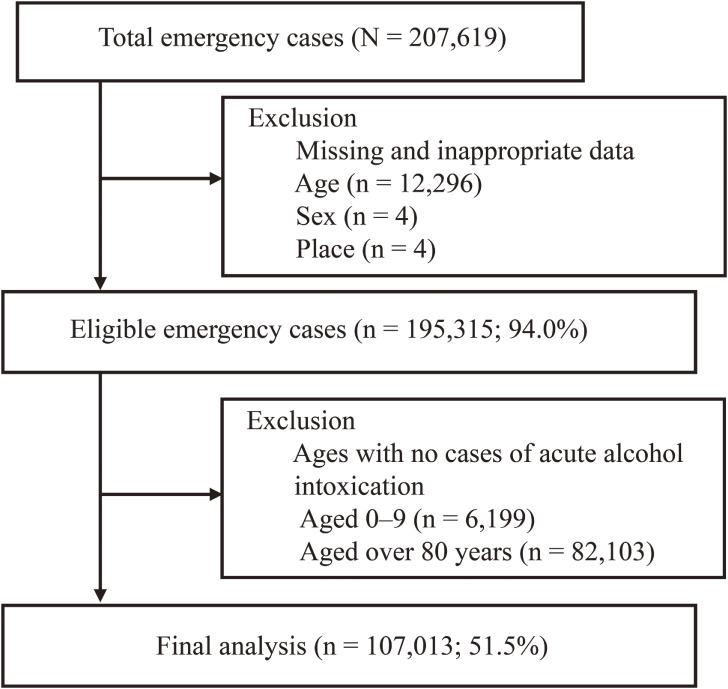
Sampling flowchart

The database contained transportation information on 207,619 patients collected from 2019 to 2023, of which 12,304 were excluded because of missing data on patient age (*n* = 12,296), sex (*n* = 4), or departure site (*n* = 4). We also excluded patients aged 0–9 (*n* = 6,199) and >80 years (*n* = 82,103) because none of them had acute alcohol intoxication. Thus, we analyzed data from 107,013 patients (Fig. 1).

To ensure patient confidentiality, we anonymized all data in accordance with the Kochi Prefecture’s medical policy, and the process was supervised by the Kochi prefectural government. The study protocol was approved by the Ethical Review Committee of the Kochi University School of Medicine (approval number #2020-116; approval date: 25 November 2020) and adhered to the ethical standards outlined in the Declaration of Helsinki. Informed consent was not required for our study due to the anonymization of raw data.

### Data collection and measurements

The primary outcome measure was the number of emergency transportations due to acute alcohol intoxication. Note that emergency cases due to acute alcohol intoxication cannot be classified according to a disease code because they are considered cases of toxicity. Therefore, among the diagnoses listed in the database, we chose “alcohol intoxication” and defined the outcome as “emergency transportation due to alcohol intoxication.” We used the dataset to identify the location of fire stations, patient sex, age, departure site for patient, and the severity of the acute alcohol intoxication among patients who underwent emergency transportation.

### Data analysis

We calculated the descriptive statistics for acute alcohol intoxication and reported the categorical variables as numbers and percentages. We used chi-square tests to uncover the associations among location of the fire station, patient sex, age, departure site, and severity.

We then conducted multiple logistic regression to determine the outcomes of emergency transportation due to acute alcohol intoxication. We used data from 2019 as reference to assess the differences in the number of transportations throughout the year. To establish the representativeness of our pre-COVID-19 baseline data (2019), we compared the number of acute alcohol intoxication cases from 2017 and 2018. In 2017, the total number of emergency transportations in 2017 was 40,131, among which 403 (0.9%) were attributed to acute alcohol intoxication; these numbers for 2018 were 41,306 transportations and 424 cases (1.02%), respectively. These figures demonstrate consistency in both the overall number of emergency transportations and the proportion of cases related to acute alcohol intoxication in the two years preceding our baseline study period. These comparative data are not presented in tabular form in this paper.

We adjusted our analyses for department (Kochi City as reference), sex (female as reference), age (older than 70 years as reference), departure site (home as reference), and severity (mild as reference). Statistical significance was defined as a two-tailed *p*-value < 0.05. We performed all analyses using Stata/MP software (version 16.0; StataCorp).

## Results

**Fig. 2:**
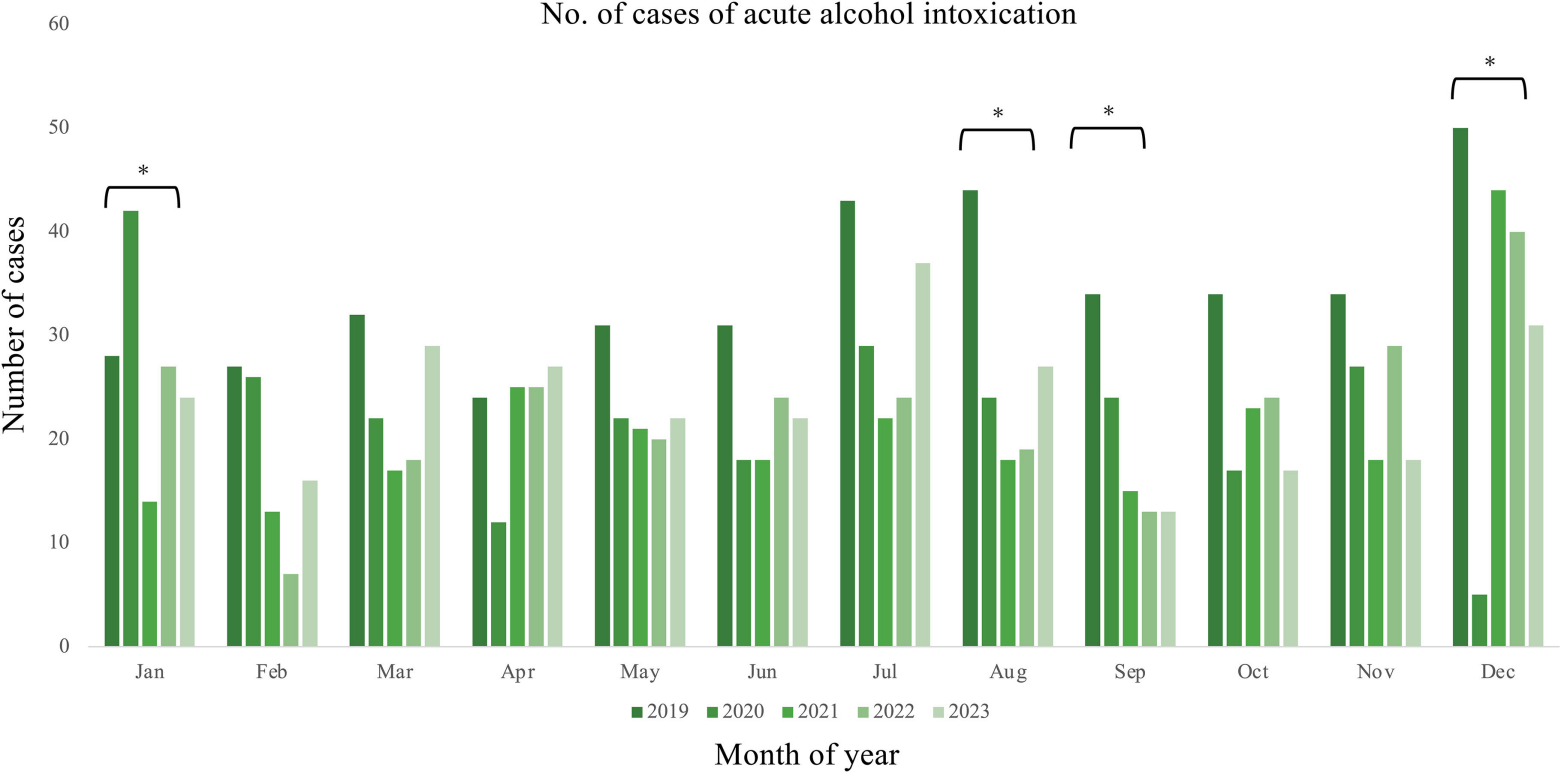
Number of cases of acute alcohol intoxication by month. The * symbol denotes a statistically significant difference (*p* < 0.05) in the monthly emergency transport volume for acute alcohol intoxication cases between the years being compared.

Figure 2 shows the number of emergency transportations due to acute alcohol intoxication during each month for the period of 2019–2023. The lowest number of transportations due to acute alcohol intoxication occurred in December 2020, while the highest number occurred in December 2019.

Regarding the number of transportations by month, we noted significant differences between December and January, which are the year-end and New Year months, and August and September, when many summer events usually occur (Fig. 2).

Table 1 presents the demographics of the emergency transportation cases from 2019 to 2023 (*p* < 0.01). Approximately 1.5% of all cases were related to acute alcohol intoxication. The number of emergency transportation cases due to acute alcohol intoxication declined (vs. 2019) to 268 (1.3%), 248 (1.2%), 270 (1.2%), and 283 (1.3%) in 2020, 2021, 2022, and 2023, respectively. Moreover, age group trends showed a decrease in emergency transportations for the 10–19 age group in 2020 and 2021.

**Table 1:** Demographics of all cases of emergency transportation according to year

**Variables**	**All years**	**2019**	**2020**	**2021**	**2022**	**2023**	***p* ^a^**
	**107,013**	**22,437 (21.0)**	**19,997 (18.7)**	**20,279 (19.0)**	**22,088 (20.6)**	**22,212 (20.8)**	
**Department**							0.56
Kochi City	50,357 (47.1)	10,637 (47.4)	9,417 (47.1)	9,581 (47.3)	10,311 (46.7)	10,411 (46.9)	
Other	56,656 (52.9)	11,800 (52.6)	10,580 (52.9)	10,698 (52.8)	11,777 (53.3)	11,801 (53.1)	

**Sex**							0.73
Male	59,627 (55.7)	12,436 (55.4)	11,216 (56.1)	11,293 (55.7)	12,286 (55.6)	12,396 (55.8)	
Female	47,386 (44.3)	10,001 (44.6)	8,781 (43.9)	8,986 (44.3)	9,802 (44.4)	9,816 (44.2)	

**Age group**							<0.01
10–19	6,828 (6.4)	1,423 (6.3)	1,205 (6.0)	1,249 (6.2)	1,514 (6.9)	1,437 (6.5)	
20–29	7,020 (6.6)	1,539 (6.9)	1,223 (6.1)	1,283 (6.3)	1,501 (6.8)	1,474 (6.6)	
30–39	6,534 (6.1)	1,482 (6.6)	1,213 (6.1)	1,257 (6.2)	1,269 (5.8)	1,313 (5.9)	
40–49	9,624 (9.0)	1,998 (8.9)	1,914 (9.6)	1,885 (9.3)	1,933 (8.8)	1,894 (8.5)	
50–59	13,406 (12.5)	2,829 (12.6)	2,533 (12.7)	2,516 (12.4)	2,793 (12.6)	2,735 (12.3)	
60–69	21,006 (19.6)	4,719 (21.0)	4,075 (20.4)	3,925 (19.4)	4,140 (18.7)	4,147 (18.7)	
70–79	42,595 (39.8)	8,447 (37.7)	7,834 (39.2)	8,164 (40.3)	8,938 (40.5)	9,212 (41.5)	

**Departure site**							<0.01
Home	60,760 (56.8)	12,120 (54.0)	11,311 (56.6)	11,634 (57.4)	12,735 (57.7)	12,960 (58.4)	
Hospital	10,145 (9.5)	2,124 (9.5)	1,940 (9.7)	2,064 (10.2)	2,011 (9.1)	2,006 (9.0)	
Older adult care facility	2,758 (2.6)	479 (2.1)	492 (2.5)	544 (2.7)	654 (3.0)	589 (2.7)	
Other^b^	33,350 (31.2)	7,714 (34.4)	6,254 (31.3)	6,037 (29.8)	6,688 (30.3)	6,657 (30.0)	

**Severity**							<0.01
Mild	54,411 (50.9)	11,584 (51.6)	9,883 (49.4)	10,147 (50.0)	11,512 (52.1)	11,285 (50.8)	
Moderate	36,199 (33.8)	7,431 (33.1)	6,933 (34.7)	7,020 (34.6)	7,320 (33.1)	7,495 (33.7)	
Severe	13,249 (12.4)	2,904 (12.9)	2,640 (13.2)	2,575 (12.7)	2,664 (12.1)	2,466 (11.1)	
Dead	1,459 (1.4)	254 (1.1)	266 (1.3)	286 (1.4)	338 (1.5)	315 (1.4)	
Other	672 (0.6)	139 (0.6)	145 (0.7)	116 (0.6)	123 (0.6)	149 (0.7)	
Missing	1,023 (1.0)	125 (0.6)	130 (0.7)	135 (0.7)	131 (0.6)	502 (2.3)	

**Acute alcohol intoxication**	1,481 (1.4)	412 (1.8)	268 (1.3)	248 (1.2)	270 (1.2)	283 (1.3)	<0.01

^a^Chi-square test.

^b^The other emergency transportation departure sites include shops, restaurants and other catering organizations, public places, and roads.

Table 2 shows the demographic characteristics of emergency transportation cases due to acute alcohol intoxication from 2019 to 2023. Emergency transportations due to acute alcohol intoxication were more common in the prefectural capital (i.e., Kochi City, 72.3%) than in other areas of Kochi Prefecture (*p* < 0.01), among men (66.5%) than among women (33.5%, *p* < 0.01), most common among those aged 20–29 years (37.6%, *p* < 0.01), and most cases involved a mild severity (76.6%, *p* < 0.01).

**Table 2:** Demographics of emergency transportations due to acute alcohol intoxication

**Variables**	**All**	**Acute alcohol intoxication**	**No^a^**	***p* ^b^**
		**n (%)**		
		**1,481 (1.4)**	**105,532 (98.6)**	
**Year**				<0.01
2019	22,437 (21.0)	412 (27.8)	22,025 (20.9)	
2020	19,997 (18.7)	268 (18.1)	19,729 (18.7)	
2021	20,279 (19.0)	248 (16.8)	20,031 (19.0)	
2022	22,088 (20.6)	270 (18.2)	21,818 (20.7)	
2023	22,212 (20.8)	283 (19.1)	21,929 (20.8)	

**Department**				<0.01
Kochi City	50,357 (47.1)	1,070 (72.3)	49,287 (46.7)	
Other	56,656 (52.9)	411 (27.8)	56,245 (53.3)	

**Sex**				<0.01
Male	59,627 (55.7)	985 (66.5)	58,642 (55.6)	
Female	47,386 (44.3)	496 (33.5)	46,890 (44.4)	

**Age group**				<0.01
10–19	6,828 (6.4)	51 (3.4)	6,777 (6.4)	
20–29	7,020 (6.6)	557 (37.6)	6,463 (6.1)	
30–39	6,534 (6.1)	191 (12.9)	6,343 (6.0)	
40–49	9,624 (9.0)	182 (12.3)	9,442 (9.0)	
50–59	13,406 (12.5)	193 (13.0)	13,213 (12.5)	
60–69	21,006 (19.6)	170 (11.5)	20,836 (19.7)	
70–79	42,595 (39.8)	137 (9.3)	42,458 (40.2)	

**Departure site**				<0.01
Home	60,760 (56.8)	344 (23.2)	60,416 (57.3)	
Hospital	10,145 (9.5)	4 (0.3)	10,141 (9.6)	
Older adult care facility	2,758 (2.6)	0 (0.0)	2,758 (2.6)	
Other	33,350 (31.2)	1,133 (76.5)	32,217 (30.5)	

**Severity**				<0.01
Mild	54,411 (50.9)	1,135 (76.6)	53,276 (50.5)	
Moderate	36,199 (33.8)	340 (23.0)	35,859 (34.0)	
Severe	13,249 (12.4)	6 (0.4)	13,243 (12.6)	
Dead	1,459 (1.4)	0 (0.0)	1,459 (1.4)	
Other	672 (0.6)	0 (0.0)	672 (0.6)	

^a^Emergency transportations for reasons other than acute alcohol intoxication.

^b^Chi-squared test.

Table 3 shows the demographics of patients transported because of acute alcohol intoxication by year. We observed significant changes in age and departure site over the years; in 2020, the number of transportations among those aged 10–19 years increased. Among those aged 20–29 years, the number of transportations decreased in 2020 (33.6%) and 2021 (31.1%) compared with 2019 (43.0%). The number of moderate severity cases decreased year by year, while the number of mild severity cases increased year by year.

**Table 3:** Demographics of emergency transportations due to acute alcohol intoxication by year

**Variables**	**All**	**2019**	**2020**	**2021**	**2022**	**2023**	***p* ^a^**
	**n (%)**						
	**1,481**	**412 (27.8)**	**268 (18.1)**	**248 (16.8)**	**270 (18.2)**	**283 (19.1)**	
**Department**							0.63
Kochi City	1,070 (72.3)	307 (74.5)	191 (71.3)	171 (69.0)	197 (73.0)	204 (72.1)	
Other	411 (27.8)	105 (25.5)	77 (28.7)	77 (31.1)	73 (27.0)	79 (27.9)	

**Sex**							0.96
Male	985 (66.5)	268 (65.1)	178 (66.4)	167 (67.3)	182 (67.4)	190 (67.1)	
Female	496 (33.5)	144 (35.0)	90 (33.6)	81 (32.7)	88 (32.6)	93 (32.9)	

**Age group**							<0.01
10–19	51 (3.4)	11 (2.7)	17 (6.3)	6 (2.4)	10 (3.7)	7 (2.5)	
20–29	557 (37.6)	177 (43.0)	90 (33.6)	77 (31.1)	104 (38.5)	109 (38.5)	
30–39	191 (12.9)	57 (13.8)	30 (11.2)	34 (13.7)	27 (10.0)	43 (15.2)	
40–49	182 (12.3)	54 (13.1)	22 (8.2)	34 (13.7)	34 (12.6)	38 (13.4)	
50–59	193 (13.0)	39 (9.5)	36 (13.4)	39 (15.7)	40 (14.8)	39 (13.8)	
60–69	170 (11.5)	43 (10.4)	39 (14.6)	22 (8.9)	37 (13.7)	29 (10.3)	
70–79	137 (9.3)	31 (7.5)	34 (12.7)	36(14.5)	18 (6.7)	18 (6.4)	

**Departure site**							0.05
Home	344 (23.2)	78 (18.9)	78 (29.1)	62 (25.0)	67 (24.8)	59 (20.9)	
Hospital	4 (0.3)	0 (0.0)	0 (0.0)	1 (0.4)	1 (0.4)	2 (0.7)	
Other	1,133 (76.5)	334 (81.1)	190 (70.9)	185 (74.6)	202 (74.8)	222 (78.5)	

**Severity**							<0.01
Mild	1,135 (76.6)	292 (70.9)	190 (70.9)	185 (74.6)	226 (83.7)	242 (85.5)	
Moderate	340 (23.0)	119 (28.9)	77 (28.7)	62 (25.0)	42 (15.6)	40 (14.1)	
Severe	6 (0.4)	1 (0.2)	1 (0.4)	1 (0.4)	2 (0.7)	1 (0.4)	

^a^Chi-square test.

Emergency transportations due to acute alcohol intoxication decreased significantly from 2020–2023 (vs. 2019; crude odds ratio [cOR]: 0.73, 95% confidence interval [CI]: 0.62–0.85; cOR: 0.66, 95% CI: 0.56–0.78; cOR: 0.66, 95% CI: 0.57–0.77; cOR: 0.69, 95% CI: 0.59–0.80 in 2020, 2021, 2022, and 2023, respectively). Even after adjusting for confounding factors, a significant decrease prevailed during this period (adjusted odds ratio [aOR]: 0.79, 95% CI: 0.68–0.93; aOR: 0.74, 95% CI: 0.63–0.87; aOR: 0.72, 95% CI: 0.61–0.84; aOR: 0.77, 95% CI: 0.66–0.90 in 2020, 2021, 2022, and 2023, respectively). Compared with other regions, Kochi City (aOR: 2.36, 95% CI: 2.10–2.65) had a significantly higher number of emergency transportations due to acute alcohol intoxication; the same applies to men compared with women (aOR: 1.65, 95% CI: 1.47–1.84). Regarding age, there were significant differences across age groups; those aged 20–29 years showed the highest rate of emergency transportations due to acute alcohol intoxication (aOR: 12.07, 95% CI: 9.93–14.66), which was significantly higher than that of those aged 70 years and older (Table 4).

**Table 4:** Comparison of emergency patients admitted for acute alcohol intoxication from 2019 to 2023

**Variables**	**Acute alcohol intoxication**			

	**cOR**	**(95% CI)**	**aOR^a^**	**(95% CI)**
**Year**				
2019	ref		ref	
2020	**0.73**	**(0.62–0.85)**	**0.79**	**(0.68–0.93)**
2021	**0.66**	**(0.56–0.78)**	**0.74**	**(0.63–0.87)**
2022	**0.66**	**(0.57–0.77)**	**0.72**	**(0.61–0.84)**
2023	**0.69**	**(0.59–0.80)**	**0.77**	**(0.66–0.90)**
**Department**				
Kochi City			**2.36**	**(2.10–2.65)**
Other			ref	
**Sex**				
Male			**1.65**	**(1.47–1.84)**
Female			ref	
**Age group**				
10–19			0.97	(0.70–1.34)
20–29			**12.07**	**(9.93–14.66)**
30–39			**4.97**	**(3.97–6.22)**
40–49			**3.39**	**(2.70–4.25)**
50–59			**2.90**	**(2.32–3.62)**
60–69			**2.00**	**(1.59–2.51)**
70–79			ref	
**Departure site**				
Home			ref	
Hospital			**0.10**	**(0.04–0.26)**
Older adult care facility			Empty	
Other			**3.91**	**(3.44–4.43)**
**Severity**				
Mild			ref	
Moderate			0.88	(0.77–1.00)
Severe			**0.05**	**(0.02–0.11)**
Dead			Empty	
Other			Empty	

aOR, adjusted odds ratio; CI, confidence interval; cOR, crude odds ratio.

Bold text indicates significant differences.

^a^Adjusted for department, sex, age, departure site, and severity.

## Discussion

In this study, we tested the hypothesis of whether emergency transportations due to acute alcohol intoxication would return to pre-COVID-19 levels after the prolonged COVID-19 pandemic ended and normal events resumed. However, compared with the 412 cases in 2019, the number of emergency transportations due to acute alcohol intoxication reduced consistently after the COVID-19 outbreak. Furthermore, compared with the 248 cases in 2021 (i.e., the year that the number of transportations shrank the most), the number increased by 22 in 2022 and 35 in 2023. Before the COVID-19 pandemic, the number of emergency transportations due to acute alcohol intoxication was on the rise every year [23, 24]. The pandemic seems to also have had complex effects on alcohol intoxication cases and severity, as there was a decrease in moderate severity cases since 2021—that is, following the onset of the pandemic. However, research directly comparing changes in alcohol intoxication severity before and after the pandemic remain limited, highlighting the need for further investigation to fully understand this trend. Our findings also show a higher incidence of emergency transportations due to acute alcohol intoxication among men than women, a trend that remained consistent throughout the duration of the COVID-19 pandemic, suggesting its non-significant influence in the sex-based distribution of these cases. This finding underscores the need for targeted interventions that address the underlying causes of acute alcohol intoxication, particularly among men, irrespective of external factors (e.g., epidemics and pandemics).

Regarding seasons, the number of emergency transportations due to acute alcohol intoxication increased markedly during Christmas and at the end of the year, when parties are held. In the context of emergency transportations due to acute alcohol intoxication, intoxicated patients can engage in assault, verbal abuse, incontinence, and other disruptive behaviors toward medical personnel at the medical institution of destination [25]. There are also cases wherein patients refuse treatment at the medical institution of destination, and such patients may often become a problem for the whole ED [24].

In serious alcohol intoxication cases, it is necessary to call an ambulance for the patient’s emergency transportation. Notwithstanding, if the number of emergency transportations due to acute alcohol intoxication increases substantially during specific periods (e.g., the winter or at nights on weekends, when the number of transportations customarily increase), EDs may become overwhelmed. This renders reducing the number of acute alcohol intoxication cases an important endeavor for public health in Japan. To this end, it is necessary to cultivate drinking habits [16] that do not lead to emergency medical care, especially among the younger generation in Kochi Prefecture, which has a strong drinking culture. According to the 2020 census data for Kochi City, there were 26,897 individuals in the 20–29 age group, while the 30–39 and 40–49 age groups consisted of 32,609 and 47,083 individuals, respectively. In our sample, those aged 20–29 years exhibited the highest rates of acute alcohol intoxication. Thus, albeit this age group may not be the largest in Kochi city, young adults, particularly those in their twenties, may be at the highest risk of acute alcohol intoxication. Regardless of size, the disproportionately high incidence highlights the need for targeted interventions and prevention strategies to address acute alcohol intoxication in this age group.

We believe that the fact that emergency transportations due to acute alcohol intoxication did not return to pre-COVID-19 pandemic levels in the analyzed period may be associated with the decline in drinking behavior among youngsters in the region. In Europe and the United States of America, the concept of “sober curious” [26]—a lifestyle in which people who can drink dare not drink or drink only small amounts of alcohol—is gaining ground. In fact, in Japan, there has been a 26% decline in drinking compared with the peak numbers registered 30 years ago [27]. Moreover, the percentage of people in their 20s who have a drinking habit is just under 80% according to recent statistics, which is a very low figure compared to that of other generations (e.g., those in their 40s), and younger generations are drinking less and less often [28]. The reasons for the youngsters’ shift away from alcohol include growing risk aversion and recreational activity diversification. It may be that their perceptions of the benefits of drinking (e.g., getting a drink, feeling exhilarated, and being able to communicate more easily) are outweighed by its drawbacks, such as the negative effects on health, time, and money, and the risk for liver failure due to intoxication.

Another possibility is associated with the spread of social networking services, which afford people environments wherein they can communicate without having to meet and drink. Recent years have also seen various non-alcoholic and low-alcohol drinks emerge and spread, and the social attention placed on these drinks has also been bolstered. This increase in drinking options may have supported the trend of enjoying leisure time without drinking to the point of intoxication, and might have contributed to a drop in the number of emergency transportations due to acute alcohol intoxication. A previous study in Thailand—which, in June 2022, became the first country in Asia to legalize the use and purchase of marijuana leaves—reported that the prevalence of marijuana use decreased alcohol consumption, and that there is the need to further probe into whether the impact on alcohol consumption among the youth may influence other potentially-addictive and intoxicating behaviors (e.g., drug use) [29].

One factor that influences drinking behavior among youngsters is the image of drinking. A secondary analysis of ad airtime data from five free Japanese TV networks in the Tokyo area that aired between 12 August and 3 November 2019 indicates that young people watched TV more frequently during the day, and that the number of liquor ads aired during daytime was 2–3.2 times greater than that during other times of the day [30]. Furthermore, some liquor products are still being advertised despite industry self-imposed restrictions [30]. As young people are exposed to such advertisements, they may unknowingly develop an image of alcohol consumption. For instance, a survey conducted among Taiwanese high school students in 2022 concluded that youth exposure to influencer marketing of non-alcoholic beer increased their likelihood of consuming it, which in turn increased their likelihood of purchasing and consuming alcohol [31]. Thus, young people were shown to be susceptible to the influence of numerous media, including social networking services and TV, underpinning the potential future need to use various media to encourage correct drinking behavior in this demographic.

Once patients incur acute alcohol intoxication once, they may be repeatedly transported to the ED because of a greater potential for recurring episodes of intoxication [32], making early intervention a necessity for these patients. A Swiss randomized controlled trial for a smartphone intervention conducted among college students with unhealthy alcohol use (i.e., who self-reported unhealthy alcohol use during follow-up) found that the reduction in the average amount of alcohol consumed was more pronounced in the intervention than in the control group [33]. However, in the rural Japanese city where we conducted this study, there is no system in place to link patients with acute alcohol intoxication to regular treatment. Therefore, although the COVID-19 pandemic was somehow associated with a reduction in the number of acute alcohol intoxication cases, some patients (over 200 per year) still require alcohol intoxication prevention educational interventions.

A particular strength of the current study is the use of a large dataset comprising more than 100,000 data points and its inclusion of all emergency transport cases from the Kochi-Iryo-Net database during the period under scrutiny. Nevertheless, there are some limitations that warrant the reader’s attention. First, it is plausible that some individuals who experienced acute alcohol intoxication were not transported to the ED via ambulance, potentially rendering the data unrepresentative of all acute alcohol intoxication cases in Kochi Prefecture. However, given the urgency inherent in alcohol intoxication situations, it is likely that most instances were captured, including those involving fire stations in the targeted region. Second, the reliance on transportation information provided by ambulance crews introduces a source of potential reporting error; for instance, various descriptions have been used to report on acute alcohol intoxication, such as “acute alcohol intoxication” and “neurologic intoxication due to acute alcoholism.” Nevertheless, we reaffirm that some level of accuracy about departure sites and other relevant details was maintained as crew members entered this information following well-established protocols. The use of a tablet-based system, primarily developed by Kochi Prefecture, also facilitates the real-time collection and storage of emergency transportation information. Third, the exclusive reliance on transportation records from Kochi Prefecture limits the generalizability of the results to other prefectures. Nevertheless, Kochi Prefecture has unique cultural nuances regarding alcohol consumption, rendering the examinations in this study valuable for the field. While this study was focused on Kochi City and other areas within Kochi Prefecture, future studies could collect more detailed regional data, data on the occupation of transported patients, and their age. We also invite future studies conducting simultaneous examinations of trends in other prefectures along with those in Kochi Prefecture. This comparative approach would provide a more comprehensive understanding of acute alcohol intoxication patterns across different regions in Japan, allowing for a broader assessment of the generalizability of our findings.

Fourth, the absence of data on patients who declined hospital transportation after contacting emergency services and the lack of information on patient prognosis are notable gaps in our dataset, and we could not clearly pinpoint cases where transported patients initially suffered from chronic alcoholism. Fifth, our data and analyses do not discriminate patients at the individual level, implying that there may have been cases where the same person was transported many times. Sixth, we did not adjust for some important confounding factors of acute alcohol intoxication, such as sleep, anxiety, and smoking, owing to data-related constraints. This suggests the need for future studies considering individual background factors in their investigations.

In summary, the occurrence of acute alcohol intoxication cases during periods of social self-restraint underscores the ongoing need for treatment and preventive measures. These measures should afford a comprehensive approach that extends beyond acute cases and encompasses broader issues associated with alcohol consumption.

## Conclusions

We examined changes in emergency transportations due to acute alcohol intoxication after the end of the COVID-19 pandemic, when events and other activities became normalized. We found a slight increase in 2022 and 2023 compared with 2021—the year when transportations reached the lowest numbers. This study suggests that the drinking behaviors of people in Kochi Prefecture changed after the COVID-19 pandemic. This shift may have been associated with various cultural and social factors and perhaps has, to some extent, helped establish the idea that people can drink alcohol but are not forced to do so. Future research directions include investigating the social changes observed in problematic drinking behavior, especially acute alcohol intoxication, among young people.

## Abbreviations

aOR: adjusted odds ratio
CI: confidence interval
cOR: crude odds ratio
COVID-19: coronavirus disease 2019
ED: emergency department

## Declarations

### Availability of data and materials

The data that support our findings are available from Kochi Prefecture (at marina.minami@kochi-u.ac.jp) upon reasonable request, but will not be made and are not publicly available.
